# The Use of Phononic Crystals to Design Piezoelectric Power Transducers

**DOI:** 10.3390/s17040729

**Published:** 2017-03-31

**Authors:** Silvia Ronda, José Luis Aragón, Elvira Iglesias, Francisco Montero de Espinosa

**Affiliations:** 1 ITEFI-CSIC, Serrano 144, 28006 Madrid, Spain; silvia.ronda@csic.es (S.R.); eiglesia.jml@gmail.com (E.I); 2 Centro de Física Aplicada y Tecnología Avanzada, Universidad Nacional Autónoma de México, Apartado Postal 1-1010, Querétaro 76230, Mexico; aragon@fata.unam.mx

**Keywords:** phononic crystals, piezoelectric transducers, physiotherapy

## Abstract

It was recently proposed that the lateral resonances around the working resonance band of ultrasonic piezoelectric sandwich transducers can be stopped by a periodic array of circular holes drilled along the main propagation direction (a phononic crystal). In this work, the performance of different transducer designs made with this procedure is tested using laser vibrometry, electric impedance tests and finite element models (FEM). It is shown that in terms of mechanical vibration amplitude and acoustic efficiency, the best design for physiotherapy applications is when both, the piezoceramic and an aluminum capsule are phononic structures. The procedure described here can be applied to the design of power ultrasonic devices, physiotherapy transducers and other external medical power ultrasound applications where piston-like vibration in a narrow band is required.

## 1. Introduction

Ultrasonic piezoelectric power transducers normally work in the length mode and are made of two sections: a piezoceramic resonator, as the active part, and either one or two different metallic sections bonded or fixed by a screw (pre-stressed transducers). From these, the Langevin type [1] is the most common design, where the transducer length is usually half the wavelength (*λ*) at the resonance frequency. A slightly different design is that of the power transducers used for physiotherapy, which are made with two sections: the piezoceramic resonator and a metallic capsule [2]. Piezoceramics and metals with low mechanical losses are used to optimize energy delivery at the working frequency.

Langevin type transducers are mainly used when a clean length resonance with a piston-like mechanical displacement at the emitting transducer surface is required. To achieve this, a length-to-radial aspect ratio higher than 2 should be used to avoid any coupling between length and radial resonance modes and to optimize the transducer electromechanical coupling coefficient [1].

In this work, we are interested in transducers for physiotherapy, where the typical design consists of a piezoceramic disc resonator and an attached metal case that will be in contact with the human body. The simplest device can be a piezoceramic disc bonded to a metallic disc. Most of these transducers operate at discrete frequencies in the range 1–3 MHz, thus the desired length-to-radial aspect ratio to produce no interference between the thickness and the radial modes cannot be achieved since the required transducer acoustic aperture is bigger than the transducer length. The transducer aperture, and in consequence the transducer diameter, is typically in the range between 10 mm and 30 mm, and the thickness should be lower than 5 mm. These acoustic constraints imply a low length-to-radial aspect ratio, thus radial modes always interfere with the main length mode. In this case the transducer emitting surface is far from the ideal piston-like behavior [2].

Although, as it has already been mentioned, this piston-like behavior can be achieved by adequately controlling the geometry of the resonators, it would be desirable to find a way to achieve this regardless the geometry and dimensions of the resonator. Piezocomposite structures have been proposed since the 80s to improve the electromechanical efficiency of the piezoelectric resonators by decreasing the specific acoustic impedance of the piezoceramics [3]. The most successful piezocomposite design consists of dicing a piezoceramic resonator along its thickness, forming a square pattern with grooves filled with a polymer. By controlling the width and pitch of the dice, lateral resonances in the planar direction are strongly attenuated, the surface vibrates in-phase and larger electromechanical coefficients are attained. Also, triangular-cut piezocomposites have been designed for unimodal operation [4]. Piezocomposites are the best option for ultrasonic imaging applications where low acoustic power is used and large frequency bandwidth is required. However, they are not useful for ultrasonic high power applications at narrow frequency band, which is our main interest in this work. The basic requirement of a piezocomposite design is to fix the spacing of the bars away from one half the wavelength.

Recently we proposed the application of the principles behind photonic and phononic materials to attenuate or even stop the lateral resonances, producing in this way a piezoceramic resonator adequate for operation in the thickness mode with an in-phase vibration surface [5]. Phononic materials prevent propagation of waves in certain frequency ranges by making use of the fundamental properties of waves, such as scattering and interference, which can produce band gaps, that is, a range of frequencies within which waves cannot propagate through the structure [6,7,8,9,10,11]. The dispersion relation of the radial waves coming from the lateral contour of a transducer can be modified by making a periodic array of holes along the longitudinal main vibration direction such that these radial waves cannot propagate. Thus, contrary to the usual requirement of piezocomposite design, in this case the strict Bragg condition is required, so the diffracting elements must be at half a wavelength. It should be said that in the field of piezoelectric resonators, phononic crystals where the periodic array consists of piezoelectric elements embedded on a substrate were first studied [12,13,14]. A different approach consisting of a piezoelectric material with a periodic repetition of hollow cylinders was used to study surface acoustic waves in these piezoelectric phononic crystals [15]. 

Here, we use the phononic crystal design proposed in [15] and compare the performance of different transducer designs, looking for the most adequate for physiotherapy applications, that is, in terms of mechanical vibration amplitude and acoustic efficiency. We study transducers consisting of a periodic array of cylindrical voids drilled along the main vibrating direction in either one or both the transducer component sections: the piezoceramic and the metallic (aluminum) capsule. We tested four designs and concluded that that the best design for physiotherapy applications is obtained when both, the piezoceramic and an aluminum capsule are phononic structures. For clinical applications the design where only the piezoceramic is a phononic structure could also be adequate because the emitting face (capsule) has no holes.

## 2. Materials and Methods

**Figure 1:**
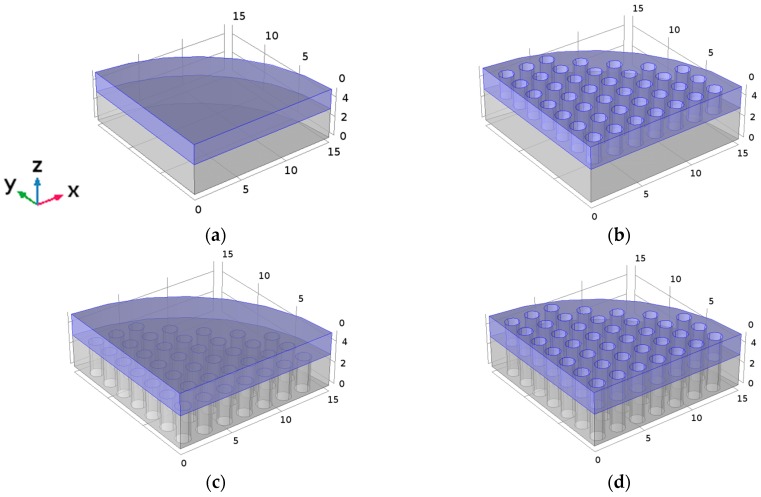
Geometry of the piezoelectric transducer used for the simulations. The scale is in mm; the piezoceramic PZT-4 disc is in blue and the aluminum plate in grey. (**a**) Model 1 and reference mechanical axis; (**b**) Model 2; (**c**) Model 3 and (**d**) Model 4.

The materials used to prepare the samples were PZT-4 piezoceramic as active element and aluminum as passive acoustic line. Four different transducer designs were built. Model 1 consists of a piezoceramic PZT-4 disc with thickness *t*_c_ = 2 mm and radius *r*_c_ = 15 mm bonded to an aluminum disc with thickness *t*_Al_ = 3 mm and the same radius. With these dimensions, and the thickness condition, the transducer should vibrate at 1 MHz. In Model 2 both transducer sections have the same dimensions, but the commercial piezoceramic is transformed into a phononic structure by drilling a square lattice of holes in the piezoceramic disc, with a lattice parameter *a* = 2 mm and holes of 1.3 mm diameter [2]. Model 3 is the same as Model 1, but with a square lattice of holes drilled only in the aluminum disc (not in the piezoceramic); the lattice parameter and filling factor used are the same as in Model 2. Finally, Model 4 consists of a transducer where both, the piezoceramic and the aluminum discs are phononic structures with the same design as in Model 2. Geometries of the four models can be found in Figure 1, where the *z* axis is the piezoceramic polarization direction. Throughout this paper, the *z* axis will be the out-of-plane vibration direction.

These models were studied numerically and experimentally. For the numerical study, the piezoelectric devices were simulated first using finite elements with COMSOL Multiphysics^®^ 4.4, using the PZT-4 and the aluminum material properties included in the COMSOL library. The calculated magnitudes were the module of the input electrical impedance, the phase of the piezoceramic and the out-of-plane vibration mechanical amplitude of the emission face (in this case, the bottom face of the aluminum).

Experimental measurements of the input electrical impedance were carried out with a 4294A Impedance Analyzer (Agilent, Santa Clara, CA, USA) and the out-of-plane mechanical amplitudes were recorded using laser vibrometry. The vibrometry experimental setup consisted of an OFV 50000 single-point vibrometer (Polytec, Waldwronn, Germany) with VD06 and DD900 decoders with a OFV 505 head, a set of UT 100-75 linear stages (Micro-Controle, Newport Co., Irvine, CA, USA) with a Micro-Controler TL78 main frame, a 3000 Wave Surfer oscilloscope (Teledyne LeCroy, Heidelberg, Germany,) and homemade software to control the stages and the signal acquisition.

## 3. Results

In a phononic crystal consisting of a square array of air holes, the lattice parameter of the perforations must be around half the wavelength of the wave to be diffracted and the band-gap width is proportional to the so-called filling factor π(*r*^2^/*a*^2^), where *a* is the lattice parameter and *r* is the radius of the perforation [5]. When a transducer is made of different materials as it is the case of the transducers studied here, the optimal lattice parameter is different for each transducer section. Aluminum has propagation velocities at different propagation modes (bulk or plate), around 1.5 times the corresponding to PZT. That means that both the lattice parameter and the hole diameter should be 1.5 times bigger for the same frequency. In Ref. [5] a PZT-5A piezoelectric disc with 20 mm of diameter and 2 mm of thickness was studied. There, it was shown that with a lattice parameter *a* = 2 mm and holes of 0.7 mm radius (38% of filling factor), clear indications of the filtering of radial modes at the resonance frequency existed. With these lattice parameters, the band gap in aluminum should be centered at a frequency around 1.5 MHz. Nevertheless, if we use these results, where a filtering band close to 700 kHz around the thickness resonance was measured, we expected a good degree of filtering of the radial modes in the aluminum plate around 1 MHz. 

### 3.1. FEM Modelling

To compare with experimental results, a frequency range from 300 kHz to 1.2 MHz was used for all calculations. In the four models, the bottom piezoelectric disc face was grounded and the upper face was excited with an electric potential of 1V. A mechanical damping was introduced, as isotropic structural factor, of 1 + 2 × 10^−2^*i*. Given the symmetry of the perforated disc, it is enough to consider only a quarter of the disc, provided that symmetry conditions are applied. The typically sampling size (the finite element mesh size) to solve a wave propagation problem should be at least five elements per wavelength. In the calculated models, bulk and plate waves (mainly symmetric Lamb modes) propagate. Velocities of cylindrical waves coming from the contour of the PZT and aluminum plates with 2 mm and 3 mm of thickness are around 4000 ms^−1^, which means a wavelength of 4mm at 1 MHz. Therefore, finite element mesh of about *h* = 1.5 × 10^−4^ m in the drilled domain and *h* = 5 × 10^−4^ m in the remaining domain was used. The mesh was even refined at the boundaries.

**Figure 2:**
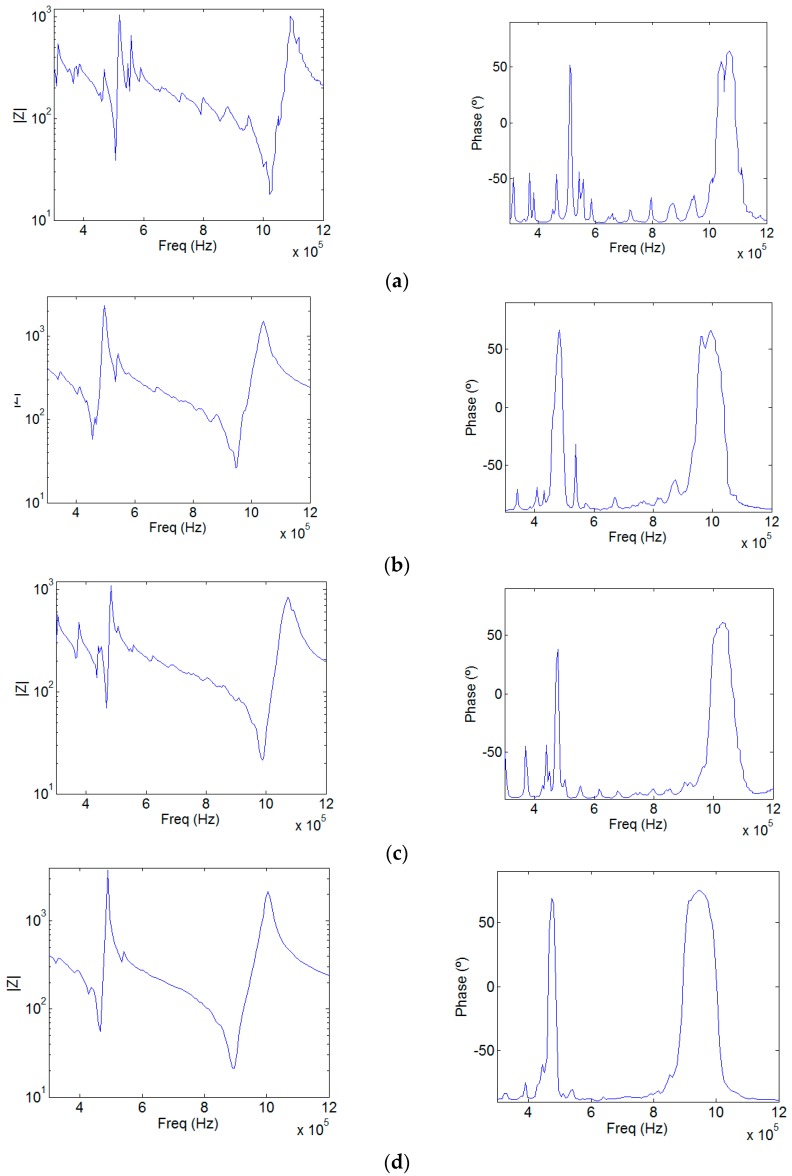
Calculated input electrical impedance. (**a**) Model 1 impedance module in logarithmic scale and phase. The same for Model 2 (**b**), Model 3 (**c**) and Model 4 (**d**).

Model 1 (Figure 1a) where neither the piezoceramic or the aluminum section have perforations, is the typical commercial design of a 1 MHz physiotherapy transducer. Figure 2a shows its simulated module input electrical impedance and its phase, calculated using a frequency step of 4.66 × 10^3^ Hz (about 193 points of the used frequency range). The first minimum of the impedance module is at 500 kHz and the second minimum is at 1 MHz, the first and second thickness resonances related to half and one wavelength, respectively.

**Figure 3:**
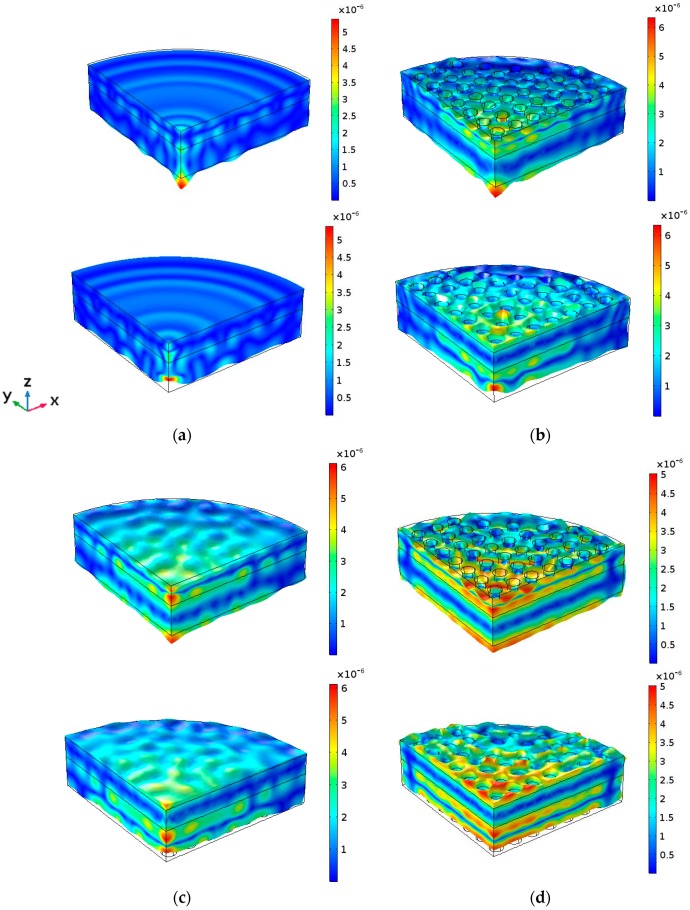
*Z* direction vibration amplitude calculated results for the four models at their respective main thickness resonance (one wavelength resonance). (**a**) Maximum elongation time position of the aluminum face (**up**) and minimum elongation time position (**bottom**) of Model 1. The same for Model 2 (**b**), Model 3 (**c**) and Model 4 (**d**). The excitation voltage of the piezoceramic disc in all the models was 1V. The bar scale is in mm. The frequency for each model was 997 kHz, 951 kHz, 988 kHz and 894 kHz, respectively.

**Figure 4:**
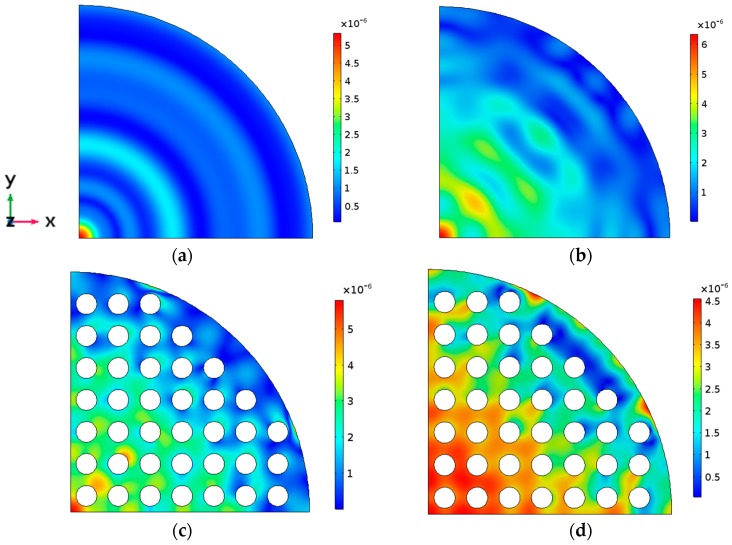
The z direction vibration amplitude calculated at the aluminum surface for the four models at their respective main thickness resonance (one wavelength resonance). (**a**) Model 1, (**b**) Model 2, (**c**) Model 3 and (**d**) Model 4. The excitation voltage of the piezoceramic disc in all the models was 1V. The bar scale is in mm. The frequency for each model was 997 kHz, 951 kHz, 988 kHz and 894 kHz, respectively.

These two main thickness resonances are obviously coupled with all the radial modes of the entire cylindrical structure. The plate modes excited at the cylindrical contour interfere with the thickness mode, thus modifying the amplitude and phase of the out-of-plane displacement of both the PZT and the aluminum transducer faces. The signature of a combination of Bessel functions are superimposed to the plane wave in-phase displacement. The z component of the cylindrical waves is not so strong as to create complete counter-phase vibration. The consequence of this mixture of thickness and radial vibration modes is that the mechanical displacement amplitude pattern of the main circular surfaces of the transducer at thickness resonance (997 kHz) becomes far from the thickness in-phase assumption, showing the signature of radial modes both in the amplitude and in the phase, as shown in Figure 3a and Figure 4a. Consequently, the acoustic field emitted into a medium, such as the human body, is not related to the acoustic diffraction piston-like assumption and the physiotherapists, even aware of the circular diffraction theory, will have no idea about the real field distribution emitted into the body [2].

Figure 2b shows the input electrical impedance of Model 2. In this model, only the piezoelectric disc is a phononic structure. As it can be seen, the signature of the radial modes is less evident; the more one approaches the center of the phononic crystal frequency band (1 MHz), the cleaner this signature is. Moreover, as reported in [5], the resonance frequency and the quality factor *Q* decrease. The mechanical displacement amplitude pattern of the aluminum surface at the resonance frequency (951 kHz) is shown in Figure 3b and Figure 4b. It is evident that the influence of the radial modes is lower than in the case of the device without phononic crystal. The out-of-plane vibration still has radial symmetry, but it is less concentrated and in-phase (Figure 3b). The phononic crystal structure of the piezoceramic was capable of improving the phase vibration, even at the aluminum transducer surface.

Figure 2c shows the input electrical impedance of Model 3, where only the aluminum disc is a phononic structure. The filtering of the radial modes is not as evident as in Model 2 since the design of the phononic crystal is not well fitted to the aluminum plate, as explained at the beginning of this Section. The vibration is mainly in phase and its amplitude is more regular along the surface. As expected, circular modes are not present (Figure 3c). Despite that the aluminum phononic crystal is not centered at 1 MHz, Model 3 seems to be effective in some degree. The resonance frequency is lower than the measured in Model 1 because the aluminum section suffers the same softening effect reported in [5].

**Figure 5:**
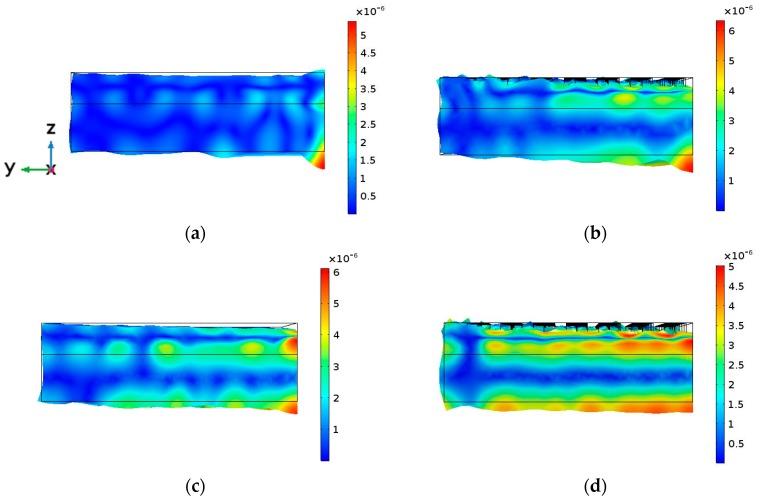
Maximum out-of-plane vibration amplitude at maximum elongation of the aluminum surface in the yz section, *x* = 0, of the transducer for 1 V excitation of the piezoceramic. (**a**) Model 1, (**b**) Model 2, (**c**) Model 3 and (**d**) Model 4. The bar scale is in mm. The frequency for each model was 997 kHz, 951 kHz, 988 kHz and 894 kHz, respectively.

Finally, concerning Model 4, where both the piezoceramic and the aluminum discs were drilled, in Figure 2d we observe that the filtering is quite clear and effective in all the frequency range studied. The softening effect is now stronger, lowering the frequency up to 890 kHz. Figure 3d and Figure 4d show that, apart from the transducer contour, all the surface vibrates in phase and with a quite regular amplitude distribution. Local maxima are located at the inter-hole space. No radial modes are observed, neither in the input electrical impedance or in the vibration pattern. Figure 5 shows the maximun out of plane vibration amplitude at the maximum elongation of the aluminum surface in the *yz* section (*x* = 0) for each transducer model at their corresponding resonance frequency (997 kHz, 951 kHz, 988 kHz and 894 kHz, respectively). All of them were excited with the same electrical amplitude signal (1 V).

**Figure 6:**
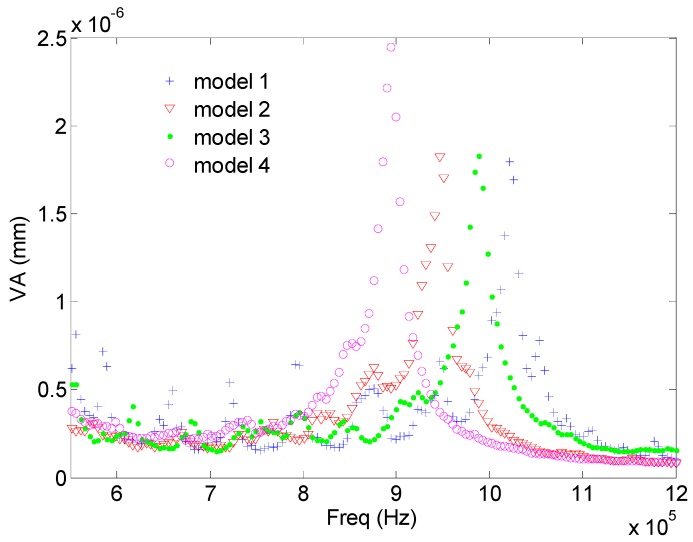
Average of the maximum value of the vibration along the *z* axis of all the calculated points in the aluminum face (VA) as a function of the vibration frequency.

**Figure 7:**
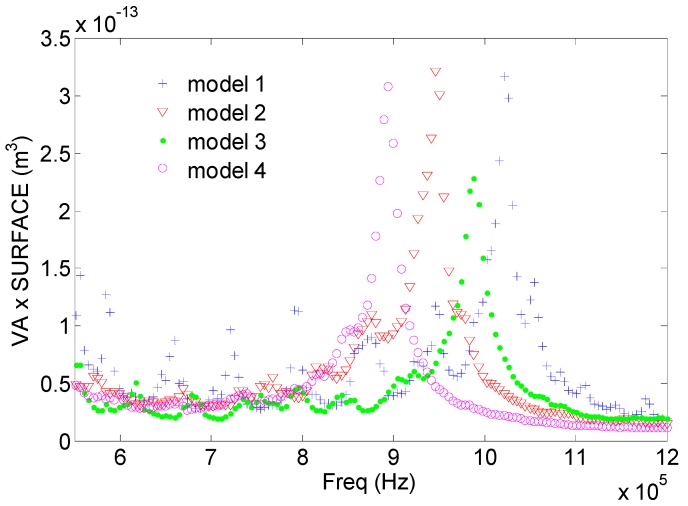
Average of the maximum value of the vibration along the *z* axis of all the calculated points in the aluminum face (VA × surface) as a function of the vibration frequency, multiplied by the real surface of each mode.

To compare the transduction efficiency of the different models, the average of the maximum value of the vibration along the *z* axis of all the calculated points in the aluminum face was calculated as a function of the vibration frequency. Figure 6 shows the average, without taking into account the effective radiating surface of each model. As observed, the highest value of each model is attained at the corresponding resonance frequency. The bandwidth is similar. Model 4 displays the highest average mechanical displacement. The softening of the resonance structure is correlated with the increase of the mechanical displacements as expected (see [5]). If the actual emitting surface is considered (Model 3 and Model 4 has a surface with holes), if the average is multiplied by the surface, then these results are modified. From Figure 7 we see that this average amplitude vibration is the same as the obtained from Models 1, 2 and 4. Since Model 4 is the only one with a homogenous phase vibration, we conclude that it is the most efficient.

Summarizing. Model 4 is the best option in terms of mechanical vibration amplitude and acoustic efficiency. Nevertheless, Model 2 is more practical for clinical applications because the emitting face has no holes. Model 2 has the same average mechanical displacement that Model 4 but the vibration distribution is less uniform in amplitude (Figure 3, Figure 4 and Figure 5).

**Figure 8:**
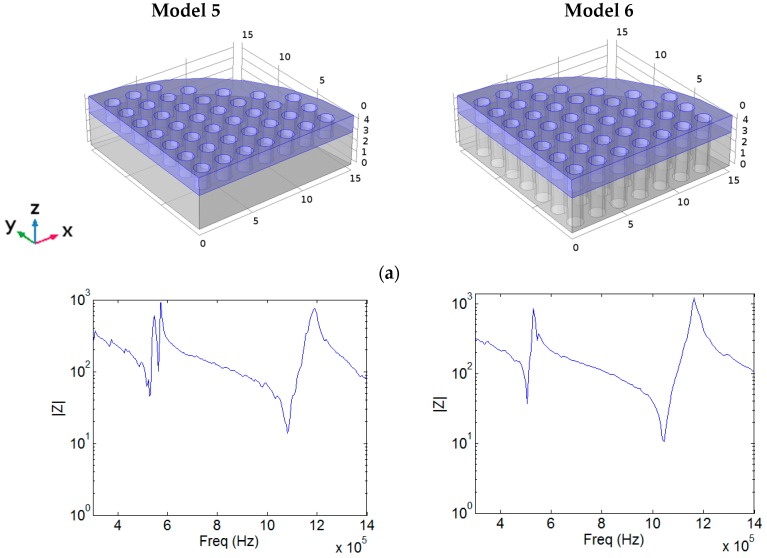

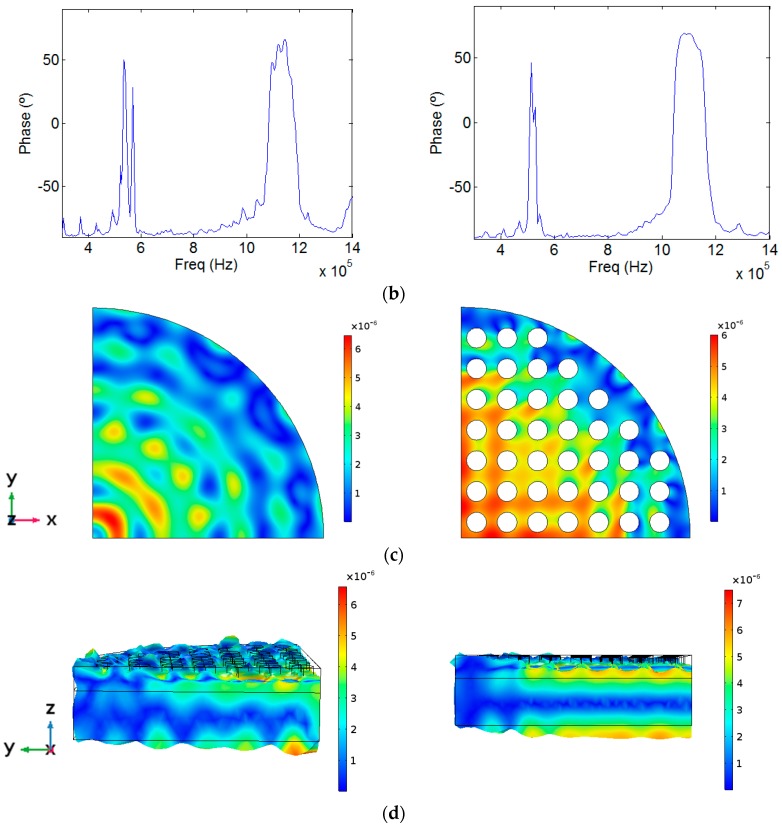
Simulations for Model 5 (left column) and Model 6 (right column) for a 1V excitation of the piezoceramic disc. Scales are in mm: (**a**) Model Setup, (**b**) Module of the input electrical impedance and phase, (**c**) Value of the out of plane component of the mechanical displacement at the aluminum surface for the resonance frequency (1.082 MHz for Model 5 and 1.042 MHz for Model 6) and (**d**) Value of the maximum out-of-plane vibration amplitude in the *yz* section of the transducers for the resonance frequency.

Since the standard lowest frequency used in physiotherapy is 1 MHz, we now consider two more models (Model 5 and 6), similar to Models 2 and 4, but where the piezoceramic disc has 1.5 mm of thickness (Figure 8a). The KLM monodimensional model was used to choose this piezoceramic thickness to get a transducer with a frequency close to the standard 1 MHz.

Figure 8 shows the module and phase of the input electrical impedance as a function of frequency, the out-of-plane component of the mechanical displacement at the aluminum surface for the resonance frequency and the value of the maximum out-of-plane vibration amplitude at maximum elongation of the aluminum surface in the yz section, for Models 5 and 6. The corresponding resonance frequencies are 1.082 MHz and 1.042 MHz, respectively. We observe that behaviour of these models is quite similar to those of Models 2 and 4.

### 3.2. Experimental Results

Ferroperm Pz26 hard piezoceramics were used to build Models 1, 3, 5 and 6. The aluminum discs and the drilling of the piezoceramics were made at our workshop. The piezoceramic drilling process was made with an automatic programmable drilling system. After several trials, a set of drilling parameters including the maximum number of holes for each drill were defined and finally used to produce the discs with holes. The piezoceramic and the aluminum sections were bonded with Araldite D & HY935. 

**Figure 9:**
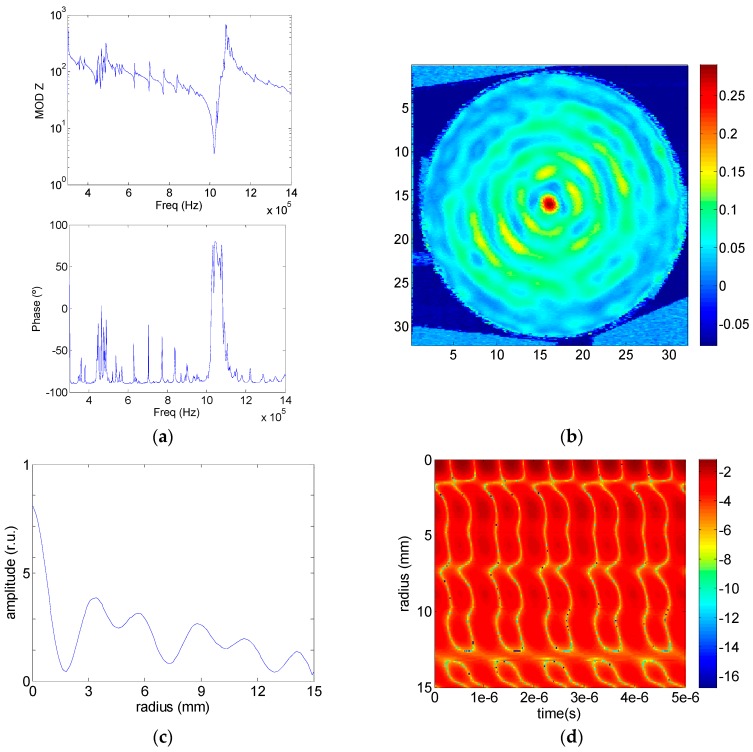
Experimental results with Model 1, (**a**) Module and phase of the electrical impedance; (**b**) Aluminum surface out-of-plane mechanical displacement in linear arbitrary units; (**c**) Mechanical displacement amplitude along the radius; (**d**) Relative mechanical displacement amplitude as a function of time along the radius in logarithmic scale.

Models 1 was first tested to compare with the numerical results. Figure 9 shows the set of electrical and mechanical outputs of Model 1. Figure 9a shows the electrical impedance module and phase and Figure 9b shows the aluminum surface out-of-plane mechanical displacement measured by laser vibrometry in linear arbitrary units. Figure 9c shows the maximum amplitude of the mechanical displacement along the radius. 

As it can be seen, the agreement with the simulated model is quite good (see Figure 2a, Figure 3a, Figure 4a and Figure 5). As the vibrometer spot is scaned from the centre to the border the maximum mechanical amplitude, the wave phase along the radius and the recorded vibration signal as a function of time, were measured and are shown in Figure 9d. The phase variation can be observed by following the amplitude variation for a given time. Horizontal axis correspond to a time of 5 μs. Bar scale gives the amplitude of the signal in relative units logarithmic scale. The figure shows that the phase of the mechanical displacemet varies along the radius as a consequence of the strong coupling between the thickness mode and the thickness component of the radial modes.

**Figure 10:**
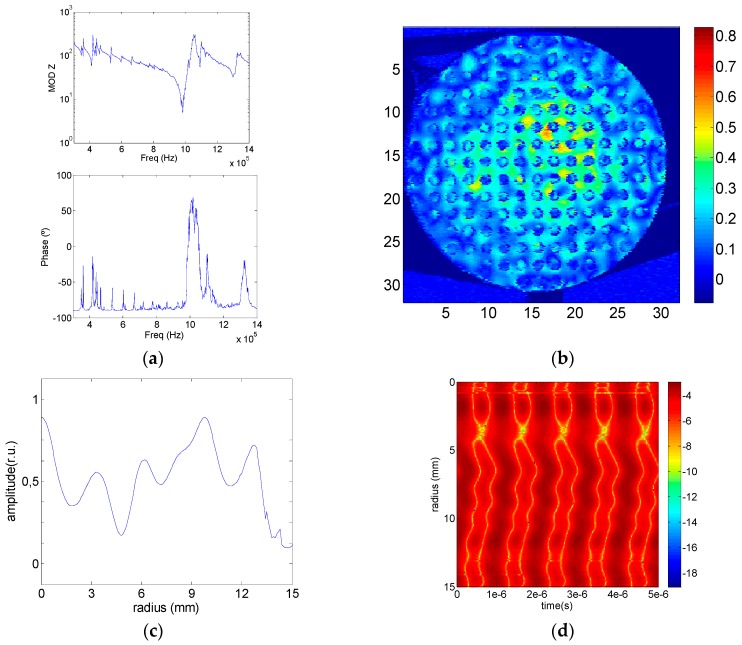
Experimental results for Model 3, (**a**) Module and phase of the electrical impedance; (**b**) Aluminum surface out-of plane mechanical displacement in linear arbitrary units; (**c**) Mechanical maximum displacement amplitude along the radius; (**d**) Relative mechanical displacement amplitude as a function of time along the radius in logarithmic scale.

**Figure 11:**
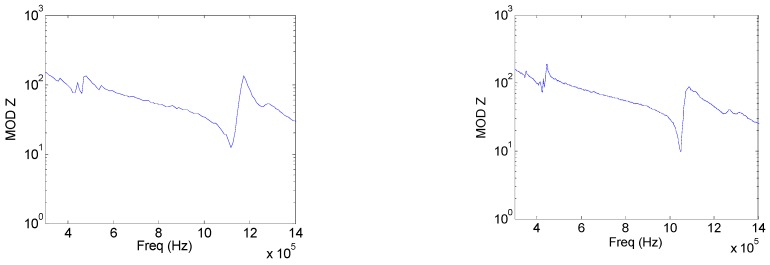

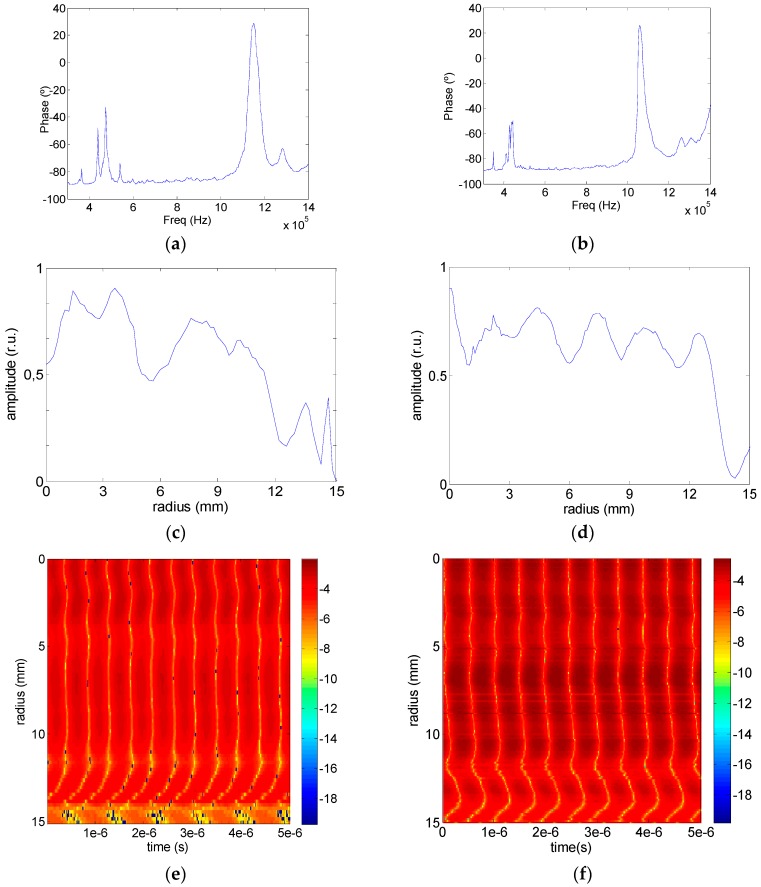
Experimental tests of Models 5 and 6; (**a**) Module and phase of the electrical impedance of Model 5; (**b**) Module and phase of the electrical impedance of Model 6; (**c**) Amplitude of the mechanical displacement along the radius of Model 5; (**d**) Amplitudeof the mechanical displacement along the radius of Model 6; (**e**) Relative mechanical displacement amplitude as a function of time, also along the radius (logarithmic scale) in Model 5; and (**f**) Relative mechanical displacement amplitude as a function of time, also along the radius (logarithmic scale) in Model 6.

Now Model 3 was tested to observe the filtering produced by the aluminum section with drilled holes. Figure 10 shows the same tests as those shown in Figure 9 for Model 1. As predicted by the numerical results, the electrical and vibration measurements show a clear filtering of the radial modes. Interestingly, the phase change observed in Figure 10d agrees with the one observed in Figure 3c. The surface pattern of Figure 10b displays the same square shadow, rotated in the 110 crystalographic direction, which is also observed in Figure 4c. Finally, Models 5 and 6 were fabricated and tested. Since the goal is to get power transducers with a vibration pattern close to the piston-like assumption, the Model 6, with a complete phononic structure, is the best model as was also predicted by the FEM model. The variation of the mechanical amplitude distribution along the radius is less than 25% along the 75% of the entire transducer emitting face (Figure 11d) with an in-phase vibration behavior (Figure 11f). Model 5 shows a 40% variation of the mechanical amplitude vibration along the radius in the 50% of the emitting surface with in–phase vibration (Figure 11c,e). As in the case of the Model 2, the Model 5 is also a possibility if a metal plate without holes is required for technical reasons.

**Figure 12:**
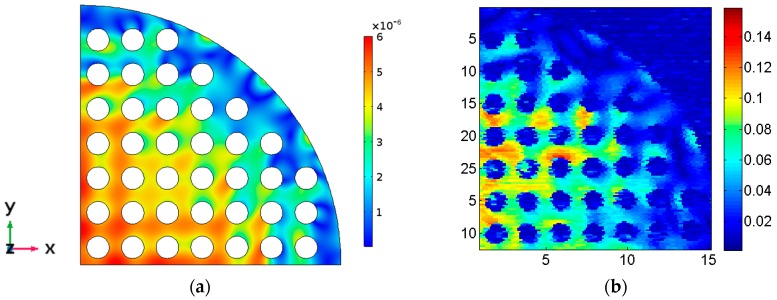
Model 6 out-of-plane mechanical displacement for the resonance frequency around 1 MHz. (**a**) FEM model, (**b**) experimental measurement in linear arbitrary units.

Finally, in Figure 12 the calculated maximum z mechanical displacement of the Model 6 is compared with the experimentally measured maximum out-of-plane mechanical displacement. The vibration distribution agrees quite well. The bar scales show that the relative values also agree.

## 4. Conclusions

Typical power ultrasonic transducer model with two sections, piezoceramic and metal, has been modified following the phononic crystal working principle. This is achieved by transforming the transducer into a phononic crystal by drilling a lattice of holes spaced by an adequate distance calculated to stop the radial modes of the resonator, that is, the resonances arising from the cylindrical contour and those coming from the holes themselves are stopped. The effect of the array of holes in one of the sections or in both sections has been studied with FEM models and tested with real transducers. Electrical and vibrational experimental tests have also been made. The effect produced by performing a lattice of holes either in one of the transducer sections (PZT and aluminum plates) or in both has been studied. Although cancelling the radial modes in the case of PZT is more effective because the design is optimum for this case, the same crystal lattice and filling ratio are used for both transducer sections. With only one section drilled with a phononic structure, a clear filtering of the radial modes is observed, but the effect is maximum when the two transducer sections are drilled. We conclude that in terms of mechanical vibration amplitude and acoustic efficiency, the most efficient design is obtained when both, the piezoceramic and an aluminum capsule, are phononic structures. However, for clinical applications the design where only the piezoceramic is a phononic structure could also be a solution because the emitting face (capsule) has no holes. The method for designing piezoelectric power transducer and testing procedure using here can be applicable to fabricate more efficient physiotherapy ultrasonic transducers with piston-like emitting surfaces.

## References

[1] Neppiras E.A. (1973). The Pre-Stressed Piezoelectric Sandwich Transducer.

[2] Iglesias E., Chinchurtreta F., De Frutos J., Montero de Espinosa F. (2016). Tailoring the efficiency of ultrasonic transducers for physiotherapy. Ferroelectrics.

[3] Smith W.A., Auld B.A. (1991). Modeling 1–3 composite piezoelectrics: thickness-mode oscillations. IEEE Trans. Ultrason. Ferroelectr. Freq. Control.

[4] Dziewierz J., Ramadas S.N., Gachagan A., O’Leary R.L., Hayward G. A 2D Ultrasonic array design incorporating hexagonal-shaped elements and triangular-cut piezocomposite. Proceedings of the 2009 IEEE International Ultrasonics Symposium.

[5] Aragón J.L., Quintero-Torres R., Domínguez-Juárez J.L., Iglesias E., Ronda S., Montero de Espinosa F. (2016). Planar modes free piezoelectric resonators using a phononic crystal with holes. Ultrasonics.

[6] Maldovan N. (2013). Sound and heat revolutions in phononics. Nature.

[7] Sigalas M.N., Economou E.N. (1992). Elastic and acoustic wave band structure. J. Sound. Vib..

[8] Kushwaha M.S., Halevi P., Dobrzynski L., Djafari-Rouhani B. (1993). Acoustic band structure of periodic elastic composites. Phys. Rev. Lett..

[9] Montero de Espinosa F., Jiménez E., Torres M. (1998). Ultrasonic band-gap in a periodic two-dimensional composite. Phys. Rev. Lett..

[10] Sánchez-Pérez J.V., Caballero D., Martínez-Sala R., Rubio C., Sánchez-Dehesa J., Meseguer F., Llinares J., Gálvez F. (1998). Sound attenuation by a two-dimensional array of rigid cylinder. Phys. Rev. Lett..

[11] Vasseur J.O., Deymier P.A., Frantziskonis G., Hong G., Djafari-Rouhani B., Dobrzynski L. (1998). Experimental evidence for the existence of absolute band gaps in two-dimensional periodic composite media. J. Phys. Condens. Matter.

[12] Wilm M., Ballandras S., Laude V., Pastureaud T. (2002). A full 3D plane-wave-expansion model for 1–3 piezoelectric composite structures. J. Acoust. Soc. Am..

[13] Wang Y.Z., Li F.M., Huang W.H., Wang Y.S. (2007). Effects of inclusion shapes on the band gaps in two-dimensional piezoelectric phononic crystals. J. Phys. Condens. Matter.

[14] Wang Y.Z., Li F.M., Kishimoto K., Wang Y.S., Huang W.H. (2009). Wave band gaps in three-dimensional periodic piezoelectric structures. Mech. Res. Commun..

[15] Laude V., Wilm M., Benchabane S., Khelif A. (2005). Full band gap for surface acoustic waves in a piezoelectric phononic crystal. Phys. Rev. E.

